# Median eminence myelin continuously turns over in adult mice

**DOI:** 10.1016/j.molmet.2023.101690

**Published:** 2023-02-04

**Authors:** Sophie Buller, Sara Kohnke, Robert Hansford, Takahiro Shimizu, William D. Richardson, Clemence Blouet

**Affiliations:** 1 Wellcome-MRC Institute of Metabolic Science and Medical Research Council Metabolic Disease Unit, University of Cambridge, Cambridge, UK; 2 Wolfson Institute for Biomedical Research, University College London, London, UK

**Keywords:** Median eminence, Hypothalamus, Oligodendrocyte, Myelin, Microglia, Nutrient sensing, ME, median eminence, OL, oligodendrocytes

## Abstract

**Objective:**

Oligodendrocyte progenitor cell differentiation is regulated by nutritional signals in the adult median eminence (ME), but the consequences on local myelination are unknown. The aim of this study was to characterize myelin plasticity in the ME of adult mice in health or in response to chronic nutritional challenge and determine its relevance to the regulation of energy balance.

**Methods:**

We assessed new oligodendrocyte (OL) and myelin generation and stability in the ME of healthy adult male mice using bromodeoxyuridine labelling and genetic fate mapping tools. We evaluated the contribution of microglia to ME myelin plasticity in PLX5622-treated C57BL/6J mice and in *Pdgfra-Cre/ER*^*T2*^*;R26R-eYFP;Myrf*^*fl/fl*^ mice, where adult oligodendrogenesis is blunted. Next, we investigated how high-fat feeding or caloric restriction impact ME OL lineage progression and myelination. Finally, we characterized the functional relevance of adult oligodendrogenesis on energy balance regulation.

**Results:**

We show that myelinating OLs are continuously and rapidly generated in the adult ME. Paradoxically, OL number and myelin amounts remain remarkably stable in the adult ME. In fact, the high rate of new OL and myelin generation in the ME is offset by continuous turnover of both. We show that microglia are required for continuous OL and myelin production, and that ME myelin plasticity regulates the recruitment of local immune cells. Finally, we provide evidence that ME myelination is regulated by the body’s energetic status and demonstrate that ME OL and myelin plasticity are required for the regulation of energy balance and hypothalamic leptin sensitivity.

**Conclusions:**

This study identifies a new mechanism modulating leptin sensitivity and the central control of energy balance and uncovers a previously unappreciated form of structural plasticity in the ME.

## Highlights

• New myelinating oligodendrocytes are continuously and rapidly produced in the adult median eminence.
• Unlike what occurs in other regions, myelinating oligodendrocytes turn over in the adult median eminence.
• Median eminence myelin plasticity regulates local microglia recruitment and microglia are required for myelin plasticity in lean mice.
• Median eminence myelination is regulated by the body’s energetic status.
• Oligodendrogenesis in the adult brain is required for the regulation of energy balance and hypothalamic leptin sensitivity.

## 1 Introduction

Oligodendrocytes (OLs) are the myelin forming cells of the central nervous system (CNS). Once thought to be exclusively generated during early postnatal life in rodents, it is now evident that new myelinating OLs continue to differentiate from a pool of oligodendrocyte progenitor cells (OPCs) in various white matter tracts of the adult brain long after developmental myelination is complete [[1], [2], [3], [4], [5], [6]]. In addition to facilitating brain repair after injury [7], new OL and myelin generation in the adult brain optimizes physiological processes including motor skill learning [8, 9] and memory processing [10, 11]. Previous studies have demonstrated that new OLs generated during adulthood are long-lived and stably integrate into existing circuits [12] where they either contribute towards the modification of existing myelinated circuits, termed myelin remodeling, or myelinate previously naked axons, termed *de novo* myelination [[13], [14], [15]]. However, the rate of OPC proliferation and differentiation, and therefore new OL generation, varies by brain region with, for example, adult oligodendrogenesis occurring at a greater rate in the corpus callosum (CC) than motor cortex [2].

The median eminence (ME) is a circumventricular organ located adjacent to the arcuate nucleus (ARC) at the base of the hypothalamus, a region critical for homeostatic functions including the regulation of energy balance. Due to its unique fenestrated vasculature, the ME maintains open communication with the circulation, allowing the release of hypophysiotropic hormones directly into the portal system, and unbuffered exposure to circulating factors for optimal monitoring of circulating cues [16]. The ME is highly plastic, undergoing cellular and structural remodelling that is critical for adaptive physiological responses to stimuli such as energy deficit [17] and the regulation of the neuroendocrine axes [18]. Although the hypothalamus is largely devoid of myelin, myelinating OLs are present in the adult ME. Here, myelin can be observed in a dense band that extends laterally across the dorsal ME, directly below the tanycytes lining the wall of the third ventricle, where it ensheathes axons of magnocellular neurones [19,20].

In the ME, OPCs proliferate more rapidly than in adjacent hypothalamic nuclei *in vivo* [6,21], and the proportion of differentiating OPCs is higher and regulated by nutritional signals [19]. However, the consequences on local myelination are unknown. The initial goal of this study was to quantify adult oligodendrogenesis in the ME compared to the CC, a well-characterised white matter tract where continuous OL generation occurs in adulthood [2]. Using bromodeoxyuridine (BrdU) and genetic fate mapping approaches, we demonstrate that new myelinating OLs are rapidly and continuously produced in the healthy adult ME, and that new OL and myelin generation are offset by the continuous turnover of myelinating OLs which maintains stable OL and myelin density in the ME over time. Mechanistically, we show that microglia are essential for the maintenance of ME OL and myelin production and that OL plasticity is required for the maintenance of the local immune cell populations. Through the assessment of myelination and OL lineage plasticity in the ME of mice exposed to a high fat diet (HFD) or caloric restriction (CR), we provide evidence that ME myelin amounts are regulated by peripheral energy availability. Finally, we provide evidence that adult generated OLs contribute to the regulation of energy balance and leptin action.

## 2 Materials and methods

### 2.1 Animals

Details of all reagents and animal models are detailed in Table 1. All animal experiments were performed in accordance with the UK Home Office regulations under the Animals (Scientific Procedures) Act (1986) and with the approval of the University of Cambridge Animal Welfare and Ethics Review Board. Animals were group-housed in a specific pathogen free facility and maintained on a standard 12-hour light/dark cycle (lights on 7:00–19:00) at 22 °C with *ad libitum* access to water and standard laboratory chow (SAFE R105, SAFE Complete Care Competence, Rosenberg, Germany) unless otherwise stated. All experiments were performed on male mice starting from postnatal day 60 (P60). C57BL/6J mice were obtained from Charles Rivers Laboratories (Saffron Walden, UK). *Pdgfrα-Cre/ER*^*T2*^*;Rosa26-YFP;Myrf*^*fl/fl*^ mice were provided by William Richardson at University College London. *Opalin-Cre/ER*^*T2*^*;Ai9* mice were provided by Professor Thora Karadottir at the University of Cambridge. *Plp-Cre/ER*^*T2*^ mice (stock number 005975) and B6; 129P2-*Mapt*^*tm2Arbr*^/J *(tau-mGFP)* mice (stock number 021162) were obtained from the Jackson Laboratories (Bar Harbour, Maine).

### 2.2 Tamoxifen preparation and administration

Tamoxifen (Sigma, St Louis, Missouri) was prepared in corn oil (Sigma) by sonication at 37 °C at 30 mg/ml prior to administration by oral gavage at 300 mg/kg on 4 consecutive days. Where tamoxifen was administered by the intraperitoneal route, tamoxifen was prepared at 20 mg/ml for injection at 80 mg/kg for 8 consecutive days.

### 2.3 Cumulative bromodeoxyuridine (BrdU) paradigm

Mice were administered BrdU (Sigma), dissolved in drinking water at 1 mg/ml continuously for up to 60 h and concomitantly administered up to two intraperitoneal injections of BrdU (50 mg/kg; prepared in sterile saline at 5 mg/ml) in any 12-hour period. Mice were sacrificed 2, 12, 24 and 60 h after the onset of BrdU administration.

### 2.4 Caloric-restriction paradigm

C57BL/6J mice were acclimatised to single-housing and randomly allocated to the *ad libitum* fed or caloric restricted (CR) group. For animals that were CR, 70% of the average amount of food eaten by the control group over the previous 24-hour period was provided at ZT11 daily. 7 days after the onset of CR, all animals were administered four intraperitoneal injections of BrdU (50 mg/kg) within a 24-hour period starting at ZT5 and ending at ZT4 on the eighth experimental day. Mice were sacrificed 1 h later, at ZT5, on experimental day 8.

### 2.5 45% High fat diet studies

C57BL/6J mice were fed a diet with 45% of calories from fat (45% HFD - D12451, Research Diets, New Brunswick, New Jersey) or a standard chow diet for 8 weeks from 7 to 8 weeks of age. Prior to culling, animals were administered 4 intraperitoneal injections of BrdU (50 mg/kg) over 24 h as described above.

For fate mapping studies in *Plp-Cre/ER*^*T2*^*;R26R-eGFP* mice, animals were fed a 45% HFD from P60 and were administered tamoxifen 1- or 8- weeks later. Mice were trans-cardially perfused 14 days after tamoxifen administration.

### 2.6 Microglia ablation with PLX5622

C57BL/6J mice were fed a control AIN-76A diet (D10001, Research Diets) or AIN-76A with PLX5622 at 12,000 parts per million (D19101002Si, Research Diets) from 8 weeks of age for 2 weeks prior to perfusion fixation.

### 2.7 Metabolic phenotyping of *Pdgfrα-Cre/ER*^*T2*^*;Rosa26-YFP;Myrf*^*fl/fl*^ mice

Metabolic phenotyping studies were performed in *Pdgfrα-Cre/ER*^*T2*^*;Rosa26-YFP;Myrf*^*fl/fl*^ mice from 3 weeks after tamoxifen administration.

#### 2.7.1 Assessment of energy balance

Body composition was analysed using a EchoMRI Whole Body Composition Analyser (EchoMRI, Houston, Texas). Promethion High-Definition Multiplexed Respirometry Cages (Sable Systems International, Las Vegas, Nevada) were used to analyse energy expenditure by indirect calorimetry, food intake and activity over 48 h in mice single-house for at least one week prior. Data collected during the first 24 h of each run was discarded to allow for acclimatisation of mice to the altered cage environment.

#### 2.7.2 Telemetric assessment of body temperature

TA-F10 Mouse Temperature Transmitters (Data Sciences International, St Paul, Minnesota) were tethered to the parietal peritoneal wall under isoflurane anaesthesia. One week after surgery, home cages were placed on PhysioTel Receiver plates (Model RPC-1; Data Sciences International) and core body temperature data was collected over a 48-hour period using PhysioTel software (Data Sciences International) with a MX2 (Data Sciences International) managing communication between the software and implants.

#### 2.7.3 Measurement of plasma leptin

Leptin was measured in plasma samples collected at ZT2 using a Mouse Plasma Leptin Kit (MesoScale Discovery, Rockville, Maryland) on a MESO Sector S600 Instrument (MesoScale Discovery).

#### 2.7.4 Leptin replacement during a fast

We used the protocol described by Morton et al. [22] to replace leptin to physiological values during a fast. Subcutaneous mini pumps with a flow rate of 0.25 μl/h (Model 1002; Alzet, Cupertino, California) were loaded with mouse recombinant leptin (R&D Systems, Minneapolis, Minnesota) prepared at 400 ng/μl in sterile 20 mM Tris–HCl pH 8.0 (Sigma) for delivery at 100 ng/h. Sterile vinyl tubing (V/3A) containing 48 μl sterile saline was attached to the minipump flow moderator. This was calculated to allow 7 days of saline delivery after surgery. Pumps were primed by incubation in sterile saline at 37 °C and were implanted between the scapula (subcutaneous) under isofluroane anaesthesia on experimental day 0. On the morning of experimental day 2, mice were housed in Promethion High-Definition Multiplexed Respirometry Cages (Sable Systems International) for 48 h. On experimental day 3, mice were fasted from ZT10 for 24 h during the period of vehicle administration. Animals were refed at ZT10 on experimental day 4 and returned to home cages. On the morning of experimental day 6, mice were returned to Promethion High-Definition Multiplexed Respirometry Cages (Sable Systems International) for a further 48 h. On experimental day 7, mice were fasted in the calorimeter from ZT10 for 24 h for analysis of energy expenditure and activity during the period of leptin administration. Animals were refed at ZT10 on experimental day 8 and rehoused in home cages.

#### 2.7.5 Leptin sensitivity test

Leptin sensitivity was assessed in single-housed mice in a crossover fashion. Mice were fasted for 24 h prior to the administration of mouse recombinant leptin (R&D Systems) prepared at 0.3 mg/ml in 20 mM Tris–HCl pH 8.5 (Sigma) or vehicle by intraperitoneal injection at 10 ml/kg of body weight. Mice were subsequently refed standard chow and food intake and body weight gain recorded over the following 24 h. The experiment was repeated one week later, with animals being administered the alternative substance to which they received on the previous week.

#### 2.7.6 Leptin-induced pSTAT3

Mice were fasted overnight from ZT10 until ZT2, after which mice were administered an intraperitoneal injection of mouse recombinant leptin (R&D Systems) prepared at 0.3 mg/ml in 20 mM Tris–HCl pH 8.5 (Sigma) at a dose of 10 ml/kg of body weight. Mice were terminally anaesthetised 30 min later for perfusion fixation and tissue collection.

### 2.8 Perfusion fixation

Mice were administered 50 ml pentobarbital (Dolethal, 200 mg/ml) by intraperitoneal injection to achieve deep anaesthesia. Mice were then trans-cardially perfused with 50 ml heparinised phosphate buffered saline (PBS) followed by 4% paraformaldehyde (PFA; Fisher Scientific, Waltham, Massachusetts) in PBS (pH 7.4) at a flow rate of 5 ml/min.

### 2.9 Immunofluorescence

Brains were post-fixed overnight at 4 °C in 4% PFA then cryoprotected in 30% (w/v) sucrose (Fisher Scientific) solution in PBS for at least 48 h prior to processing. Tissues were covered with Optimal Cutting Temperature (OCT) medium (CellPath, Newtown, UK) and sections were obtained at 30 μm on a Leica SM2010R Freezing Microtome (Leica, Wetzlar, Germany). All sections were subjected to heat-mediated antigen retrieval in 10 mM sodium citrate (pH6.5; Fisher Scientific) in distilled water for 20 min at 80 °C prior to washing 3 times in PBS. For sections immunolabelled for BrdU, tissues were then incubated with 2N hydrochloric acid (Sigma) in distilled water for 30 min at 37 °C. Sections were subsequently incubated with 0.1M sodium borate (pH 8.5; Sigma) in distilled water to neutralise the acid and then washed 3 times with PBS. However, for pSTAT3 immunolabelling sections were sequentially incubated with 1% sodium hydroxide (w/v; Fisher Scientific) and 1% hydrogen peroxide (v/v; Fisher Scientific) for 20 min, 0.3% glycine (Sigma) for 10 min and 0.06% sodium dodecyl sulphate (SDS; Sigma) for 10 min at room temperature in place of the heat mediated antigen retrieval step as described above. For all experiments, sections were blocked in normal donkey serum (NDS, Vector Biolabs, Philadelphia, Pennsylvania) diluted in PBS containing 0.3% Triton X-100 (0.3% PBST; Sigma) for 1 h prior to primary antibody incubation overnight at 4 °C. Primary antibodies were mouse anti-APC (OP80, Millipore, Burlington, Massachusetts) 1:500, rat anti-BrdU (ab6326 Abcam, Cambridge, UK) 1:200, rat anti-CD68 (ab53444 Abcam) 1:100, rabbit anti-degraded MBP (AB5864 Millipore) 1:1000, chicken anti-GFP (ab13970 Abcam) 1:1000, rabbit anti-Iba1 (Wako 019-19741, Richmond, Virginia) 1:500, rat anti-MBP (ab7349 Abcam) 1:500, rabbit anti-PDGFRα (3164 Cell Signalling Technologies, Danvers, Massachusetts) 1:500, rabbit anti-pSTAT3 (9145 Cell Signalling Technologies) 1:500 and goat anti-Sox10 (AF2864 R&D Systems) 1:50. Following primary antibody incubation, sections were washed 3 time with 0.1% PBST and incubated with appropriate fluorophore-conjugated secondary antibodies diluted 1:500 in 0.3% PBST for 2 h at room temperature. Sections were subsequently washed with 0.1% PBST and mounted to Clarity microscope slides (Dixon Science, Edenbridge, UK) under coverslips (1.0 thickness; Marienfeld, Lauda-Königshofen, Germany) with Vectashield Vibrance Mounting Medium with 4′,6-diamidino-2-phenylindole (DAPI; Vector Laboratories, Newark, California). Alexa405, Alexa488, Alexa555, Alexa594 and Alexa647 conjugates (Life Technologies, Carlsbad, California) were used as secondary antibodies.

### 2.10 Confocal microscopy and image analysis

For all experiments, slides and images were blinded to experimental condition. Sections were imaged with a Leica SP8 confocal microscope using either a 40× or 63× oil objective. Sections were imaged as z-stacks at intervals of 3.3 μm with tile scanning to obtain signal from the entire depth and area of the region of interest (ROI). Microscope settings were identical for image acquisition within each experiment. Images were analysed using Fiji software. For all analyses, Z stacks were projected into a single image and the area of ROIs were selected using the freehand tool and measured. For cell counts, the Fiji manual cell counter was used to count marker-positive cells. Where area of immunoreactivity was calculated, all images within an experiment were identically thresholded and the area fraction, limited to that threshold, measured. 3D reconstruction images were generated using Imaris software.

### 2.11 Oil red O staining

PFA-fixed tissues were cryoprotected in 30% sucrose solution (w/v in distilled water; Sigma) overnight before sectioning at 8–10 μm on a Leica CM1950 cryostat onto electrostatically charged slides. A stock solution of ORO was made up by gently heating 0.5 g ORO (Sigma) in 100 ml absolute isopropyl alcohol (Sigma) in a water bath overnight. A working solution was prepared by mixing 60 ml ORO stock solution and 40 ml 1% dextrin (w/v; Fisher Scientific) in distilled water. The working solution was allowed to stand for one day and filtered prior to use. Slides were rinsed in PBS and incubated with the ORO working solution for 20 min. Excess stain was rinsed off with distilled water and sections counterstained with haematoxylin for 20 s and blued in tap water. Coverslips were mounted with Pertex mounting medium (Pioneer Research Chemicals Ltd.).

### 2.12 Brightfield microscopy

Images were acquired with an Axioscan Z1 Slide Scanner (Zeiss) using a 20× objective.

### 2.13 Quantitative polymerase chain reaction

Fresh hypothalami were collected into RNALater solution (ThermoFisher) and stored at −20 °C until processing. RNA was extracted from tissues samples using an RNEasy Micro Kit (Qiagen, Hilden, Germany) following the manufacturer’s protocol. 100–200 ng of total RNA was reverse transcribed to cDNA using the High Capacity cDNA Reverse Transcription Kit (Applied Biosystems, Waltham, Massachusetts) according to the manufacturer’s instructions. Gene expression was assessed using either SYBR Green or TaqMan technologies on a QuantStudio 5 (Applied Biosystems). Relative gene expression was calculated by the 2ˆ(-ΔΔCt) method. Data were normalised to the housekeeping gene *Gapdh,* as the expression of this gene did not change between groups. Primers for SYBR Green based quantitative polymerase chain reaction (qPCR) are detailed in Table 2. Details of primers and probes used for qPCR with TaqMan technology are detailed in Table 3. All primers and probes were obtained from Sigma–Aldrich and commercially available TaqMan assays were obtained from Thermo Fisher.

**Table 1:** Key Resources Table.

Reagent or resource	Name	Source	Identifier	Additional information
Antibody	Alexa-Fluor® 488 AffiniPure donkey anti-chicken	Jackson Immunoresearch	Cat # 703-545-155	1:500 dilution
			RRID: AB_2340375	
Antibody	Donkey anti-goat Alexa Fluor® 488-conjugate	Life Technologies	Cat # A11055	1:500 dilution
			RRID: AB_2534102	
Antibody	Donkey anti-goat Alexa Fluor® 647-conjugate	Life Technologies	Cat # A21447	1:500 dilution
			RRID:AB_2535864	
Antibody	Donkey anti-mouse Alexa Fluor® 594-conjugate	Life Technologies	Cat # A21203	1:500 dilution
			RRID:AB_141633	
Antibody	Donkey anti-rabbit Alexa Fluor® 488-conjugate	Life Technologies	Cat # A21206	1:500 dilution
			RRID:AB_2535792	
Antibody	Donkey anti-rabbit Alexa Fluor® 555-conjugate	Life Technologies	Cat # A31572	1:500 dilution
			RRID:AB_162543	
Antibody	Donkey anti-rabbit Alexa Fluor® 594-conjugate	Life Technologies	Cat # A21207	1:500 dilution
			RRID:AB_141637	
Antibody	Donkey anti-rat Alexa Fluor® 488-conjugate	Life Technologies	Cat # A21208	1:500 dilution
			RRID:AB_141709	
Antibody	Donkey anti-rat Alexa Fluor® 594-conjugate	Life Technologies	Cat # A21209	1:500 dilution
			RRID:AB_2535795	
Antibody	Donkey anti-rat Alexa Fluor® 647-conjugate	Life Technologies	Cat # A78947	1:500 dilution
			RRID:AB_2910635	
Antibody	Chicken anti-GFP	Abcam	Cat # ab13970	1:1000 dilution
			RRID:AB_300798	
Antibody	Chicken anti-TMEM119	Synaptic Systems	Cat # 400-006	1:1000 dilution
			RRID:AB_2744643	
Antibody	Goat anti-GFP	Abcam	Cat # ab5450	1:1000 dilution
			RRID:AB_304897	
Antibody	Goat anti-Sox10	R&D Systems	Cat # AF2864	1:50 dilution
			RRID:AB_442208	
Antibody	Mouse anti-APC (clone CC1)	Millipore	Cat # OP80	1:200 dilution
			RRID:AB_2057371	
Antibody	Rabbit anti-degraded MBP	Millipore	Cat # AB5864	1:1000 dilution
			RRID:AB_2140351	
Antibody	Rabbit anti-Iba1	Wako	Cat # 019-19741	1:500 dilution
			RRID:AB_839504	
Antibody	Rabbit anti-PDGFRa	Cell Signalling Technologies	Cat # 3164	1:500 dilution
			RRID:AB_2162351	
Antibody	Rat anti-BrdU	Abcam	Cat # ab6326	1:200 dilution
			RRID:AB_305426	
Antibody	Rat anti-BrdU	BioRad	Cat # OBT-0030	1:200 dilution
			RRID:AB_2314029	
Antibody	Rat anti-CD68	Abcam	Cat # ab53444	1:100 dilution
			RRID:AB_869007	
Antibody	Rat anti-MBP	Abcam	Cat # ab7349	1:500 dilution
			RRID:AB_305869	
Chemical/compound	BrdU	Sigma	Cat # B5002	Prepared at 5 mg/ml in saline
Chemical/compound	Tamoxifen	Sigma	Cat #T5648	Prepared at 20–30 mg/ml in corn oil
Chemical/compound	Mouse Plasma Leptin Kit	MesoScale Discovery	Cat #K152BYC-2	
Chemical/compound	Corn oil	Sigma	Cat #C82276	
Chemical/compound	RNEasy Micro Kit	Qiagen	Cat # 74004	
Chemical/compound	RNAlater Solution	ThermoFisher	Cat # AM7021	
Chemical/compound	High Capacity cDNA Reverse Transcription Kit	Applied Biosystems	Cat # 4368814	
Chemical/compound	2X SYBR Green PCR Master Mix	Applied Biosystems	Cat # 4344463	
Chemical/compound	2X Taqman Universal PCR Master Mix	Applied Biosystems	Cat # 4305719	
Mouse Line	C57BL/6J	Charles Rivers	N/A	
Mouse Line	Myrf ^fl/fl^: Myrf^tm1Barr^	Jackson Laboratories	MGI 3851143	
Mouse Line	Pdgfra-CreERT2: Tg(Pdgfra-cre/ERT2)1Wdr	Richardson Lab, University College London	MGI 3832569	
Mouse Line	Mouse(Plp1-cre/ERT)3Pop	Jackson Laboratories	MGI 2450391	
Mouse Line	Opalin-CreERT2; Tg(Opalin-icre/ERT2)#Rjfl	Richardson Lab, University College London	MGI 5763107	
Mouse Line	R26R-eYFP: Gt(ROSA)26Sor^tm1(EYFP)Cos^	Jackson Laboratories	MGI 2449038	
Mouse Line	tau-mGFP: Mapt^tm2Arbr^	Jackson Laboratories	MGI 3590682	
Mouse Line	Ai9: Gt(ROSA)26Sor^tm9(CAG-tdTomato)Hze^	Jackson Laboratories	MGI 3809523	
Rodent diet	Rodent chow	SAFE	Cat # SAFE R105	
Rodent diet	45% high fat diet (HFD)	Research Diets	Cat #D12451i	
Rodent diet	AIN-76A Control Diet	Research Diets	Cat #D10001	
Rodent diet	AIN-76A with PLX5622	Research Diets	Cat #D19101002Si	
Software	Prism 9.0	GraphPad	https://www.graphpad.com/scientific-software/prism/	
Software	ImageJ (Fiji)	ImageJ	https://imagej.nih.gov/ij/index.html

**Table 2:** Sequences of primers used in SYBR Green quantitative polymerase chain reaction analysis.

Gene	Forward (5′-3′)	Reverse (5′-3′)
*Agrp*	ATGCTGACTGCAATGTTGCTG	CAGACTTAGACCTGGAACTCT
*Dio2*	TGCGCTGTGTCTGGAACAG	CTGGAATTGGGAGCATCTTCA
*Gapdh*	AGGTCGGTGTGAACGGATTTG	TGTAGACCATGTAGTTGAGGTCA
*Npy*	CTGCGCTCTGGGACACTAC	GGAAGGGTCTTGAAGCCTTGT
*Pomc*	ATGCCGAGATTCTGCTACAGT	TCCAGCGAGAGGTCGAGTTT

**Table 3:** Sequences of primers and probes used in TaqMan quantitative polymerase chain reaction analysis.

Gene	Assay ID	Forward (5′-3′)	Reverse (5′-3′)	Probe (5′-3′)
*Gapdh*	Mm99999915_g1	N/A	N/A	N/A
*Ucp1*	N/A	CCCGCTGGACACTGCC	ACCTAATGGTACTGGAAGCCTGG	AAGTCCGCCTTCAGATCCAAGGTGAAG

### 2.14 Statistical analysis

All data visualisation and statistical analysis was performed in Prism 9 Software (GraphPad). Details of statistical tests are found in figure legends. All data are presented as mean ± standard error of the mean (S.E.M).

## 3 Results

### 3.1 New oligodendrocytes are rapidly and continuously produced in the adult median eminence

**Figure 1:**
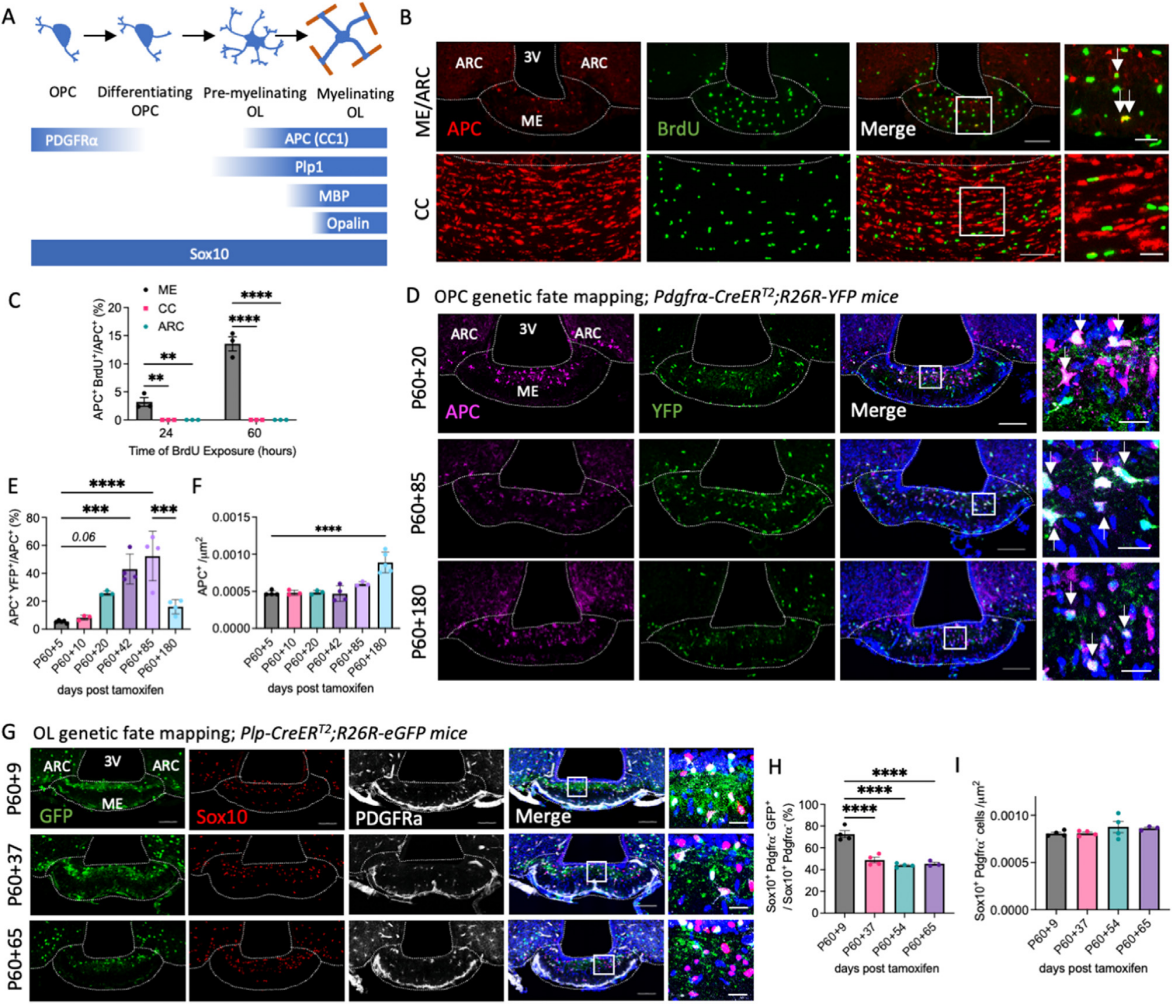
Rapid generation and turnover of oligodendrocytes in the healthy adult median eminence. (**A**) Schematic of markers used to identify oligodendrocyte lineage cells at different stages of differentiation and maturation. Representative images of OL marker and BrdU or YFP/GFP expression in the ME of (**B**) 60 hour BrdU treated C57BL/6J-, (**D**) *Pdgfra-Cre/ER*^*T2*^*;R26R–YFP -* and (**G**) *Plp-Cre/ER*^*T2*^*;R26R-eG*FP -mice and associated quantifications (**C, E, F, H, I**). For all images, overview scale bars = 100 μm, inset scale bars = 20 μm. Arrows indicate adult born OLs. Data analysed by one-way ANOVA with Dunnett’s multiple comparisons test or two-way ANOVA with Sidak’s multiple comparisons test, ∗∗p < 0.01, ∗∗∗p < 0.001, ∗∗∗∗p < 0.0001, n = 3–4/group.

We first characterised the cell cycle dynamics of OPCs *in vivo*, using a BrdU pulse-chase paradigm and immunostaining against pan-OL marker SRY-box transcription factor 10 (Sox10) and OPC marker platelet derived growth factor receptor alpha (PDGFRα) to visualise proliferative OPCs (Sox10^+^/PDGFRα^+^/BrdU^+^) in the ME, ARC and CC at postnatal day 60 (P60). In line with previous reports of rapid BrdU incorporation into ME OPCs [6,21], ME OPC total cell cycle time (T_C_) was ∼3.4 days, two to three times shorter than OPC T_c_ in the CC or ARC, (Supplementary Figure 1A,B), indicating rapid proliferation of ME OPCs. Unlike ARC and CC OPCs, ME BrdU^+^ OPCs rapidly lost PDGFRα expression (Supplementary Figure 1C) and gained expression of post-mitotic OL marker adenomatous polyposis coli (APC - also known as CC1) following 24- or 60- hours BrdU administration (Figure 1A–C), suggesting that adult-born OLs are rapidly and continuously produced in the ME.

To further establish rapid oligodendrogenesis in the adult ME *in vivo*, we labelled adult OPCs using *Pdgfrα-Cre/ER*^*T2*^*;Rosa26-YFP* mice administered tamoxifen at P60 and followed their fate over time by immunolabelling against OL markers and yellow fluorescent protein (YFP). Consistent with results from BrdU incorporation studies, labelled OPCs (Sox10^+^/PDGFRa^+^/YFP^+^) lost PDGFRa expression (Supplementary Figure 1D,E) and gained APC expression faster in the ME than in the CC, indicating rapid new OL production. Strikingly, adult-born OLs (APC^+^/YFP^+^) represented 43.0 ± 5.4% of the total OL population in the ME at P60 + 40 (Figure 1D,E) compared to just 9.4 ± 0.7% of the total OL population in the CC (Supplementary Figure 1F). Collectively, these data indicate that new OLs are rapidly and continuously produced in the adult ME.

### 3.2 Oligodendrocytes turn over in the adult median eminence

Despite continuous new OL production, histological assessments in both *Pdgfrα-Cre/ER*^*T2*^*;Rosa26-YFP* mice (Figure 1F) and a separate cohort of C57BL/6J mice (Supplementary Figure 1G–I) indicate that OL numbers are stable in the ME between P60 and P140. Intriguingly, the proportion of YFP-labelled adult-born OLs declines after P60 + 85 in the ME (Figure 1E), but not CC (Supplementary Figure 1F). This formed a rationale to directly characterise the stability of ME OLs using *Plp1-Cre/ER*^*T2*^*;Rosa26-GFP* mice, in which myelinating OLs are labelled with green fluorescent protein (GFP) following tamoxifen administration (Figure 1G). In the ME, the proportion of GFP-labelled OLs present in the ME at the time of tamoxifen administration significantly decreased between P60 + 9 and P60 + 37 and remained stable thereafter (Figure 1H) despite no differences in the total number of OLs over time (Figure 1I). Thus, myelinating OLs are short-lived and continuously replaced in the adult ME. In the CC over the same period, no decline in the proportion of GFP-labelled OLs was observed, as previously reported [12], (Supplementary Figure 1J). Consistent results were obtained with *Opalin-iCre/ER*^*T2*^*;Ai9* mice (alternative OL-specific Cre-driver; Supplementary Figure 1K–O), collectively supporting the conclusion that ME OLs turn over in adulthood.

### 3.3 Myelin is continuously replaced in the healthy adult median eminence

**Figure 2:**
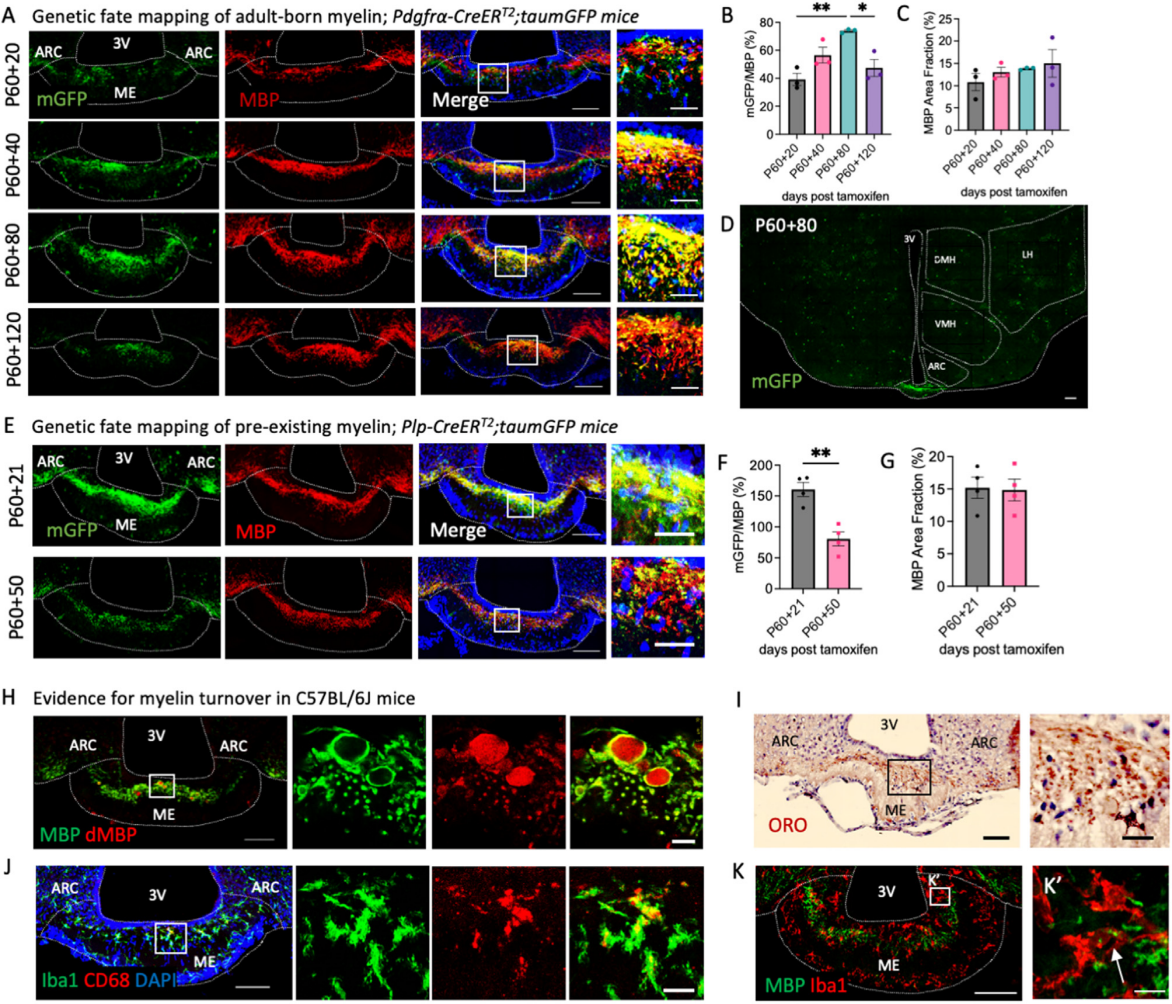
Myelin is continuously generated and replaced in the adult median eminence. Representative images of sections from (**A**) *Pdgfrα-CreER*^*T2*^*;taumGFP* and (**E**) *Plp-CreER*^*T2*^*;taumGFP* mice immunolabelled for MBP and mGFP and associated quantifications (**B, C, F, G**). Data analysed by student’s t-test or one-way ANOVA with Tukey’s multiple comparisons test, ∗p < 0.05, ∗∗p < 0.01, n = 3–4/group. (**D**) Shows an overview of mGFP signal in the hypothalamus of a *Pdgfrα-CreER*^*T2*^*;taumGFP* mouse at P60 + 80 (*NB. OPC cell bodies are labelled with a YFP reporter*). Images of ME sections from C57BL/6J adult mice immunolabelled with antibodies against MBP and degraded MBP (dMBP; **H**), oil red o (ORO; **I**) or antibodies recognising Iba1 and CD68 (**J**) or Iba1 and MBP (**K**). Arrow in (**K′**) indicates the detection of MBP immunolabelling inside a microglia expressing Iba1. For all images, overview scale bars = 100 μm, inset scale bars = 10 or 20 μm.

We next asked whether ME adult-born OLs become functionally myelinating using *Pdgfrα-Cre/ER*^*T2*^*;tau-mGFP* mice, in which tamoxifen administration at P60 induces the expression of membrane-targeted GFP (mGFP) upon OPC differentiation, resulting in the labelling of adult-generated myelin [23]. Tissues were immunolabelled for mGFP and myelin basic protein (MBP) to distinguish between adult-generated (mGFP^+^/MBP^+^) and pre-existing (mGFP^−^/MBP^+^) myelin (Figure 2A). mGFP-labelled myelin rapidly populated the ME, indicating that high rates of local OL production are associated with new myelin formation (Figure 2B), and appeared to be specific to the ME since scarce mGFP immunolabelling was detected in other hypothalamic nuclei including the dorso-medial hypothalamus (DMH), ventro-medial hypothalamus (VMH), lateral hypothalamus (LH) and ARC (Figure 2D). At P60 + 80, 73.8 ± 0.8% of ME myelin was adult-generated, but this proportion declined thereafter (Figure 2B) despite total myelin amounts remaining stable over time (Figure 2C). This suggests that in the ME, adult-generated myelin is eventually replaced by new myelin. To test this, we labelled pre-existing myelin at P60 using *Plp1-Cre/ER*^*T2*^*;tau-mGFP* mice (Figure 2E) and followed its fate over time. In the ME, the amount of mGFP-labelled pre-existing myelin decreased by ∼65% while total myelin amounts remained stable (Figure 2F,G). By comparison, previous reports demonstrate that while new myelin is generated after P60 in the CC this occurs at a slower rate and without myelin turnover [2,12]. Further supporting local myelin turnover, we observed degraded myelin (dMBP) [24] immunolabelling interspersed within the myelin band in the adult ME (Figure 2H), indicating myelin degradation. Active phagocytes (Iba1^+^/CD68^+^) as well as myelin-phagocytosing foamy macrophages are also present in the adult healthy ME (Figure 2I,J) [25]. Finally, consistent with data indicating ongoing myelin turnover in the healthy adult ME, we observed MBP immunolabelling inside Iba1+ cells in the ME of healthy adult mice (Figure 2K,L, Supplementary Video 1), suggesting active phagocytosis of myelin debris by local microglia.

### 3.4 Microglia regulate median eminence oligodendrocyte and myelin plasticity

**Figure 3:**
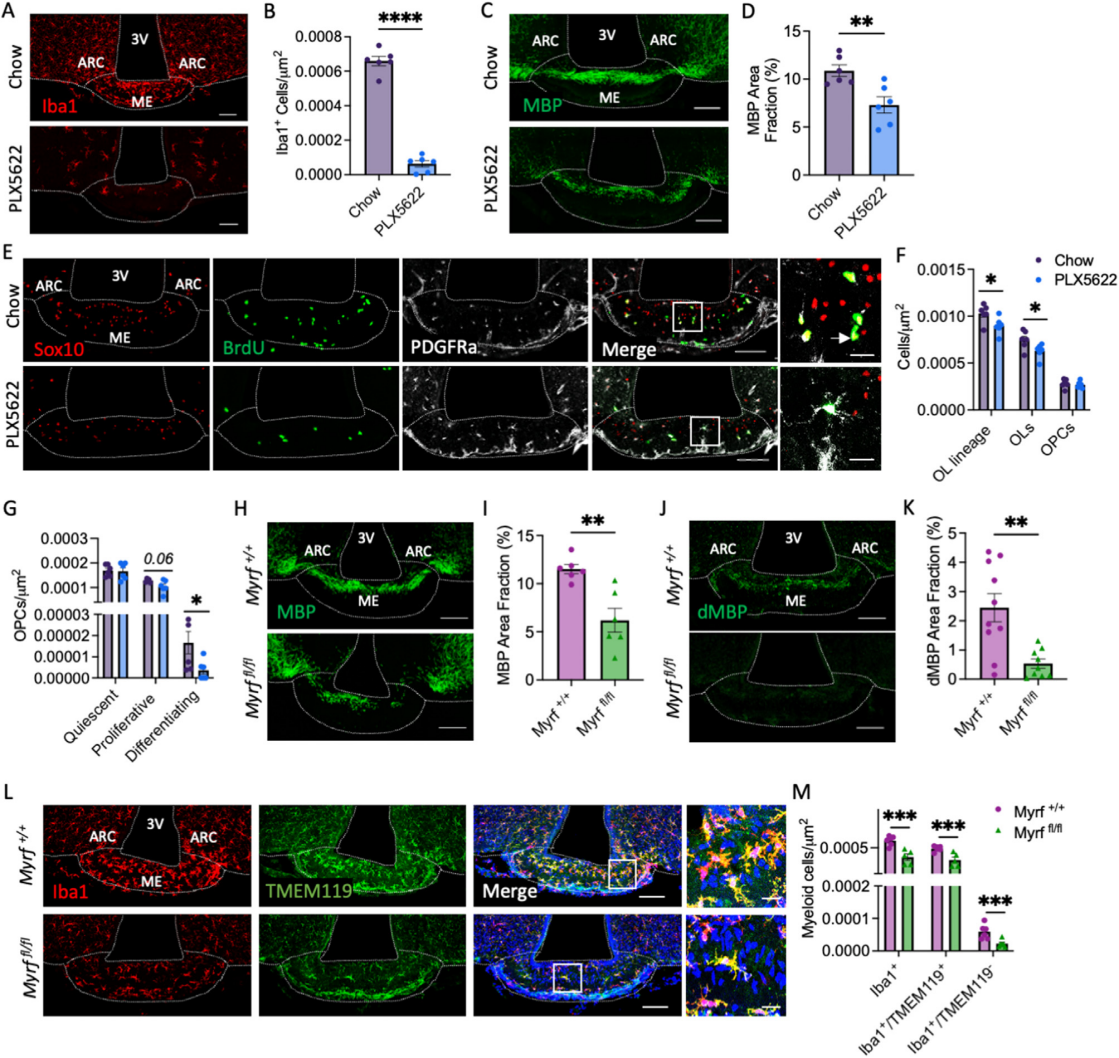
Microglia regulate median eminence oligodendrocyte and myelin plasticity. Representative images of ME sections from C57BL/6J mice fed standard chow or chow containing PLX5622 (12,000 ppm) immunolabelled with antibodies against (**A**) Iba1, (**C**) MBP or (**E**) OL markers and BrdU and associated quantifications (**B, D, F, G**). Arrow in (E) indicates a differentiating OPC (Sox10^+^/PDGFRα^−^/BrdU^+^). For (**F**) OL subtypes were distinguished as follows; OL lineage cell = Sox10^+^, OLs = Sox10^+^/PDGFRα^−^, OPC = Sox10^+^/PDGFRα^+^ and, for (**G**), Quiescent OPC = Sox10^+^/PDGFRα^+^/BrdU^−^, Proliferative OPC = Sox10^+^/PDGFRα^+^/BrdU^+^, Differentiating OPC = Sox10^+^/PDGFRα^−^/BrdU^+^. Representative images of ME sections from myelin regulatory factor (*Myrf*) knockout mice (*Myrf*^*fl/fl*^) and littermate controls (*Myrf*^*+/+*^) immunolabelled with antibodies against (**H**) MBP, (**J**) degraded MBP (dMBP) and (**L**) Iba1 and TMEM119 and associated quantifications (**I, K, M**). For all images, overview scale bars = 100 μm, inset scale bars = 20 μm. Data analysed by student’s t-test or Welch’s test, ∗p < 0.05, ∗∗p < 0.01, ∗∗∗p < 0.001, n = 6–10/group.

In demyelinating pathologies, myelin debris clearance by phagocytic microglia is critical for new OL formation and myelin repair [26]. Consequently, we hypothesised that if myelin turnover in the ME requires myelin debris removal by local phagocytes, ablation of microglia would blunt OL and myelin plasticity in the ME. To test this, C57BL/6J mice were treated with PLX5622, a colony stimulating factor 1 receptor inhibitor, to ablate microglia (Figure 3A,B). Two weeks PLX5622 treatment reduced MBP immunolabelling in the ME by ∼30% (Figure 3C,D), suggesting that local microglia are required for ME myelin maintenance. This was associated with a decrease in OPC proliferation and differentiation, and a reduction in ME OL density (Figure 3E–G). Thus, consistent with a role for local microglia in myelin turnover in healthy mice, microglia ablation decreases new OL generation in the ME. On the contrary, while PLX5622 treatment decreased microglia density in the CC (Supplementary Figure 3A), microglia ablation did not alter the density of OL lineage cells or OPC proliferation and differentiation here (Supplementary Figure 3B).

Infiltrated macrophages densely populate the ME in healthy mice [27], leading us to ask whether myelin turnover and the associated production of myelin debris is responsible for their recruitment. To test this, we used *Pdgfra-Cre/ER*^*T2*^*; R26R-eYFP; Myrf* ^*fl/fl*^ mice (*Myrf* ^*fl/fl*^ mice), where conditional deletion of myelin regulatory factor (*Myrf*), a transcription factor required for OPC terminal differentiation, blocks new OL formation in the adult brain (Supplementary Figure 2). In the ME, *Myrf* deletion rapidly reduced myelin density (Figure 3H,I), and myelin debris accumulation (Figure 3J,K) and was associated with a decrease in the density of Iba1^+^ cells (Figure 3L,M), suggesting changes in the microglial population. Since Iba1 labels both microglia and circulating macrophages, we quantified Iba1+ cells co-expressing Tmem119, a marker specific to inactivated, resting microglia. The density of both Iba1^+^/Tmem119^+^ (resting microglia) and Iba1^+^/Tmem119^-^ (activated microglia and circulating macrophages recruited to the ME) cells was significantly decreased in *Myrf* ^*fl/fl*^ mice (Figure 3L,M), indicating that adult OL formation in the ME regulates activation of microglia and/or recruitment of infiltrated macrophages. *Myrf* deletion did not affect myeloid cell density in the CC (Supplementary Figure 3C). Collectively these data indicate that (1) microglia are required for ongoing ME myelin plasticity and (2) new OL formation is necessary for the maintenance of the local myeloid cell populations and the recruitment and/or activation of microglia and peripheral macrophages.

### 3.5 Energy availability regulates median eminence myelination

Nutritional signals regulate ME OPC differentiation [19], but the long-term consequences on ME myelination are unknown. Here we tested whether energy excess or deficit produce changes in ME myelination.

**Figure 4:**
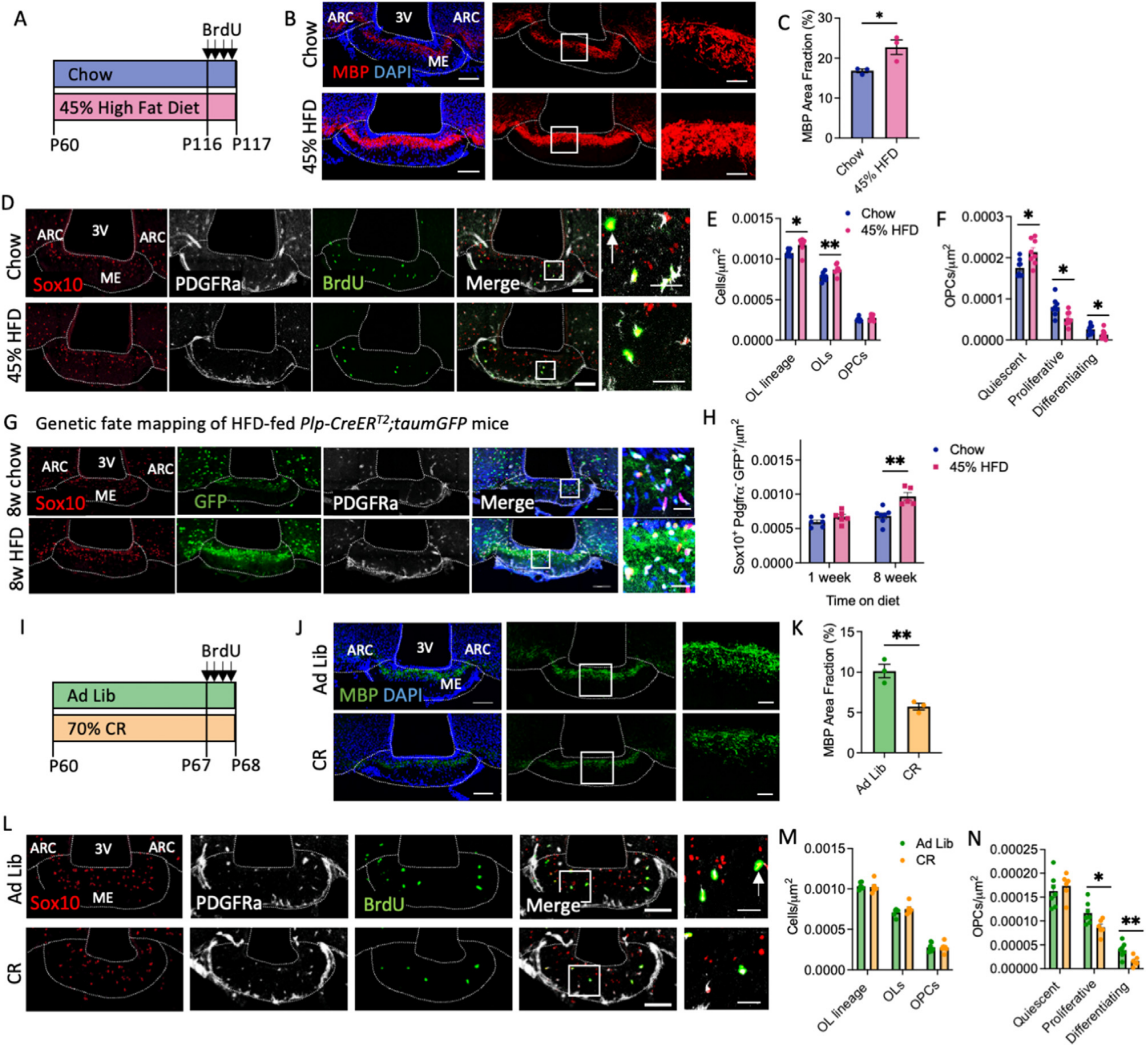
Nutritional regulation of median eminence myelination (**A**) C57BL/6J mice were fed a control diet or 45% high fat diet (HFD) for 8 weeks from postnatal day 60 (P60) and administered bromodeoxyuridine (BrdU) prior to culling. Representative ME sections from mice fed a control and 45% HFD diet immunolabelled for (**B**) MBP and (**D**) OL markers and BrdU, and associated quantifications (**C, E, F**). (**G**) Images of the ME from chow- and 45% HFD- *Plp-CreER*^*T2*^*;taumGFP* mice immunolabelled for GFP and OL markers and associated quantification (**H**). (**I**) C57BL/6J mice were fed a standard chow *ad libitum* (Ad Lib) or were 70% caloric restricted (CR) for 7 days from P60 and administered BrdU prior to culling. Representative images of ME sections from Ad Lib and CR mice immunolabelled with antibodies against (**J**) MBP and (**L**) OL markers and BrdU and associated quantifications (**K, M, N**). For all analyses, OL subtypes were distinguished as follows; OL lineage cells = Sox10^+^, OLs = Sox10^+^/PDGFRα^−^, OPC = Sox10^+^/PDGFRα^+^, Quiescent OPC = Sox10^+^/PDGFRα^+^/BrdU^−^, Proliferative OPC = Sox10^+^/PDGFRα^+^/BrdU^+^, Differentiating OPC = Sox10^+^/PDGFRα^−^/BrdU^+^. For all images, overview scale bars = 100 μm, inset scale bars = 10 or 20 μm. Data analysed by student’s t-test or two-way ANOVA with Sidak’s multiple comparisons test, ∗p < 0.05, ∗∗p < 0.01, n = 3–6/group.

We first assessed the consequences of chronic energy excess on ME myelination using a model of diet-induced obesity (DIO) (Figure 4A, Supplementary Figure 3A). DIO increased ME MBP immunolabelling (Figure 4B,C) and OL density (Sox10^+^/PDGFRα^−^; Figure 4D,E) but paradoxically decreased OPC proliferation (Sox10^+^/PDGFRα^+^/BrdU^+^) and differentiation (Sox10^+^/PDGFRα^−^/BrdU^+^; Figure 4F), suggesting that DIO blunts adult oligodendrogenesis in the ME. To test whether increased ME myelination might be the result of changes in OL turnover, we exposed *Plp1-Cre/ER*^*T2*^*; R26R-GFP* mice to the DIO paradigm. DIO significantly increased the density of YFP-labelled OLs in the ME (Figure 4G,H). indicating reduced rate of OL turnover.

Finally, we assessed ME myelination in mice exposed to a 7-day 70% caloric restriction (CR) paradigm (Figure 4I, Supplementary Figure 3B). CR reduced ME myelin amounts (Figure 4J,K) without impacting OL density (Sox10^+^/PDGFRα^−^; Figure 4L,M). However, CR decreased OPC proliferation (Sox10^+^/PDGFRα^+^/BrdU^+^) and differentiation (Sox10^+^/PDGFRα^−^/BrdU^+^; Figure 4N) in the ME. Thus, energy deficit reduces new OL production in the ME, resulting in local hypomyelination. Collectively, these data demonstrate that energy availability regulates ME myelination.

### 3.6 Adult-born oligodendrocytes are required for the regulation of energy balance and hypothalamic leptin sensitivity

OPCs have been previously implicated in the regulation of body weight and hypothalamic leptin sensing [28]. However, the contribution of adult born OLs to these processes is unknown. To determine whether adult oligodendrogenesis is required for the central control of energy balance, we assessed the metabolic phenotype of *Myrf* ^*fl/fl*^ mice between P60 and P130, a timeframe during which oligodendrogenesis is substantial in the ME but negligible by comparison in other white matter tracts [2].

**Figure 5:**
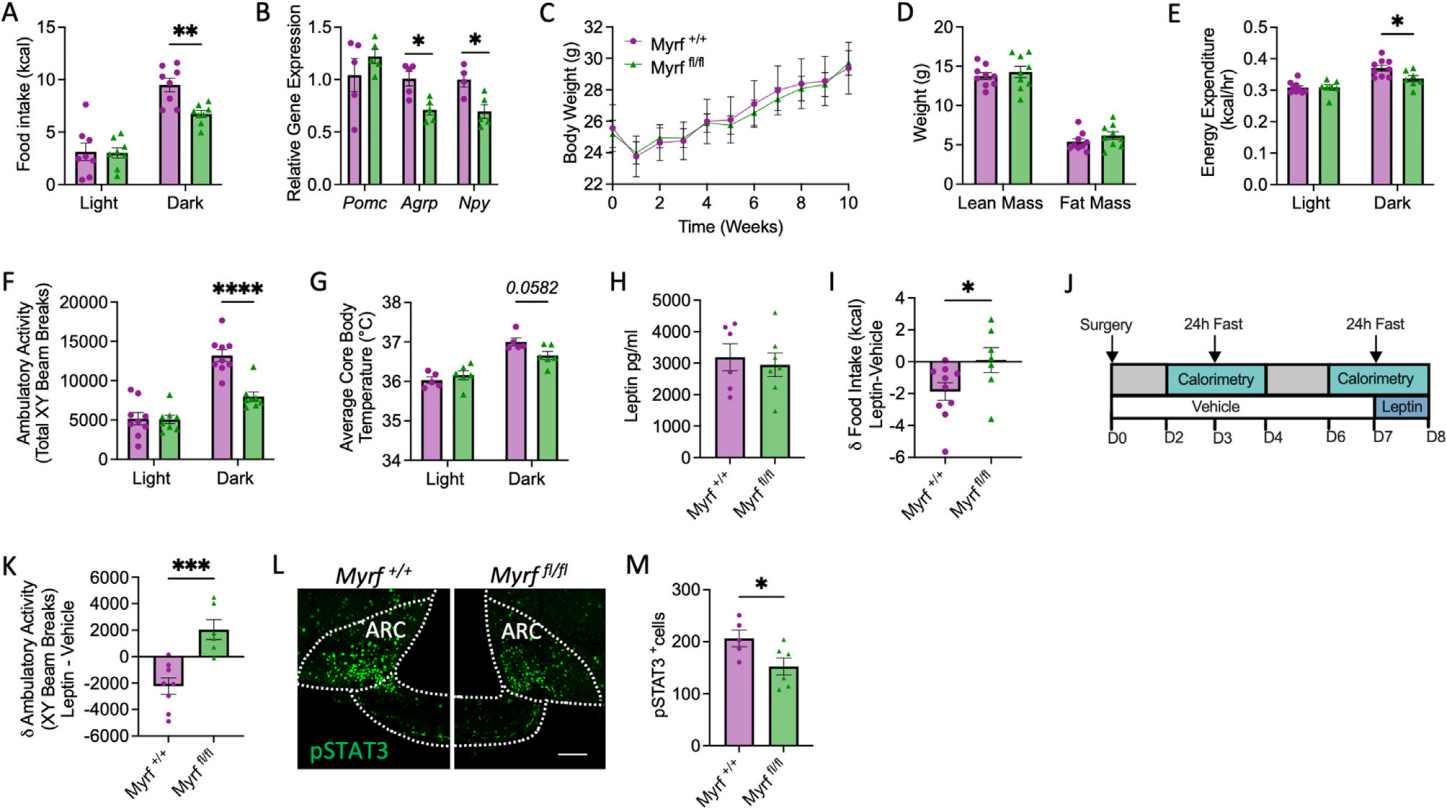
Adult born oligodendrocytes are required for the regulation of energy balance and hypothalamic leptin sensitivity. Myelin regulatory factor (*Myrf*) knockout mice (*Myrf*^*fl/fl*^) and littermate controls (*Myrf*^*+/+*^) were administered tamoxifen at postnatal day 60 to block oligodendrogenesis in the adult brain. (**A**) Light- and dark-phase food intake, (**B**) hypothalamic neuropeptide expression, (**C**) body weight, (**D**) body composition, (**E**) energy expenditure, (**F**) ambulatory activity, (**G**) average core body temperature and (**H**) plasma leptin levels of *Myrf*^*+/+*^ and *Myrf*^*fl/fl*^ mice maintained on a standard chow diet. (**I**) 24 hour food intake of *Myrf*^*+/+*^ and *Myrf*^*fl/fl*^ mice following a 24 hour fast and intraperitoneal administration of leptin (3 mg/kg) compared to vehicle. (**J**) Design of experiments assessing the physiological effects of leptin replacement (100 ng/h) during a fast in *Myrf*^*+/+*^ and *Myrf*^*fl/fl*^ mice. (**K**) Ambulatory activity of *Myrf*^*+/+*^ and *Myrf*^*fl/fl*^ mice during a fast with leptin vs. vehicle administration. (**L**) Representative images of ME sections immunolabelled for pSTAT3 following leptin administration (3 mg/kg) to 16 hour fasted *Myrf*^*+/+*^ and *Myrf*^*fl/fl*^ mice and associated quantification (**M**), scale bar represents 100 μm. Data analysed by student’s t-test or two-way ANOVA with Sidak’s multiple comparisons test, ∗p < 0.05, ∗∗p < 0.01, ∗∗∗p < 0.001, ∗∗∗∗p < 0.0001, n = 6–10/group.

Blockade of new OL production significantly reduced dark-phase food intake in *Myrf* ^*fl/fl*^ mice maintained on a chow diet (Figure 5A). Consistently, ARC expression of orexigenic neuropeptide Y (*Npy*) and agouti-related peptide (*Agrp*) was decreased following *Myrf* deletion (Figure 5B). Despite this, body weight (Figure 5C) and composition (Figure 5D) were not altered by *Myrf* deletion, presumably because energy expenditure (Figure 5E, Supplementary Figure 5A) and ambulatory activity (Figure 5F) were comparably reduced. Consistent with reduced energy expenditure, *Myrf* ^*fl/fl*^ mice defended a lower core body temperature during the dark phase (Figure 5G, Supplementary Figure 5B) and the expression of thermogenic genes deiodinase 2 (*Dio2*) and uncoupling protein 1 (*Ucp1*) was significantly reduced in the brown adipose tissue (BAT) of *Myrf* ^*fl/fl*^ mice (Supplementary Figure 5C). *Myrf* deletion had no effect on the respiratory exchange ratio (RER; Supplementary Figure 5D).

Consistent with unaltered body composition, plasma leptin levels were comparable between *Myrf* ^*fl/fl*^ mice and littermate controls (Figure 5H). However, exogenous leptin did not suppress food intake in fasted *Myrf* ^*fl/fl*^ mice (Figure 5I, Supplementary Figure 5E) and physiological leptin replacement during a fast (Figure 5J) [22] failed to produce the expected decrease in ambulatory activity in *Myrf* ^*fl/fl*^ mice (Figure 5K, Supplementary Figure 5F). Furthermore, leptin-induced pSTAT3 expression was significantly reduced in the ARC of *Myrf* ^*fl/fl*^ mice (Figure 5L,M) indicating decreased sensitivity of hypothalamic neurocircuitry to exogenous leptin. Collectively, these data support a role for OL plasticity in the physiological action of leptin.

## 4 Discussion

Adult oligodendrogenesis is emerging as a new form of brain plasticity observed in a variety of physiological contexts [8,10,11]. In the adult ME, oligodendrogenesis seems to happen within a rapid timeframe with one eighth of OLs being formed within the previous 60 hours, which is unlike what occurs in other brain regions such as the ARC and CC where no adult born OLs are detected within the same period. This high rate of new OL production in the adult ME would have little significance should these new OLs not survive and fully differentiate into myelinating OLs. However, 42 days after inducing the labelling OPCs with YFP at P60, 43% of ME OLs express YFP and hence have been formed from OPCs that differentiated after P60. This suggests that, unlike what occurs in other regions [13, 29], a large fraction of ME adult born OLs survive and become myelinating OLs. Remarkably, the high rate of new OL formation in the healthy adult ME is offset by a comparably high turnover rate, with the proportion of OLs labelled with GFP at P60 decreasing by ∼40% over 4 weeks, from 75% at P60+ 9 to 49% at P60 + 37. Collectively, using data obtained from *Plp-CreER*^*T2*^*;R26R-eGFP* and *Opalin-CreER*^*T2*^*;Ai9* mice, we estimate that the half-life of adult ME OLs is between 6 and 10 weeks which is remarkably shorter than that previously reported for CC OLs (>10 years) and sharply contrasts with evidence for OL longevity in the adult brain [12].

Our study is consistent with work by Zilka-Falb and colleagues who reported a seven-fold difference in the density of BrdU + OPCs between the ME and CC [21]. Here we found that the cell cycle time of ME OPCs is ∼3.4 days compared to ∼10.5 days in the adjacent ARC and ∼6.5 days in the CC. Of note, our estimate of OPC T_C_ in the CC is ∼3 days shorter than that previously calculated by Young and colleagues [2], which may result from different paradigms for the administration of BrdU or EdU used in our respective studies. Nevertheless, our data imply that in the healthy adult ME, each OPC produces a new OL and is replaced by a new OPC every 3.4 days, assuming that all ME OPCs proliferate [2] and undergo asymmetric division [30]. We propose that the unique cycling properties of ME OPCs are the result of the unique local environment within the ME, with exclusive unbuffered access to circulating factors including hormones, growth factors and metabolites previously reported to regulate OPCs [[31], [32], [33]]. Thus, changes in the availability of these signals with dietary challenges alters ME OL lineage plasticity.

How ME OL death is orchestrated and regulated remains to be determined but given the remarkable stability of the number of OLs in the ME, we propose that myelinating OLs may produce a homeostatic feedback signal that regulates both new OL formation and survival, allowing tight control of the OL population. Depletion of microglia impairs this homeostatic control, suggesting that they might form a necessary component of the process. In later adulthood, this homeostatic control seems to be dysregulated (starting P60 + 120 ± 6 weeks of age), with increased numbers of OLs in the ME, but whether this is due to decreased rates of turnover or increased rates of OL formation remains to be determined. The differentiation potential of OPCs might decrease with age in the ME, as has been described in the cortex and CC [2,34]. Alternatively, changes in microglial phenotypes with aging [35] might also contribute. Interestingly, high fat feeding, which rapidly induces inflammation in this area of the hypothalamus [36], blunts OL turnover and the homeostatic control of the ME OL population. Thus, the pro-inflammatory phenotype of hypothalamic microglia might reduce their ability to contribute to OL turnover.

Our work supports the functional relevance of ME OL/myelin plasticity in the regulation of energy balance. We find that in conditions of energy deficit or energy excess, OPC differentiation is blunted in the ME, albeit with opposite consequences on ME myelination. Genetically blocking adult OPC differentiation in *Myrf* ^*fl/fl*^ mice significantly changes energy intake and energy expenditure, decreases the responsiveness of ARC neurones to exogenous leptin and reduces the functional consequences of leptin. While the phenotype of *Myrf* ^*fl/fl*^ mice contrasts the hyperphagic obesity phenotype associated with OPC ablation [28], our study corroborates a role for ME OL lineage cells in hypothalamic leptin sensitivity and indicate that OPCs may play distinct roles from myelinating OLs in the regulation of energy balance. However, further work is needed to elucidate the specific role of ME myelin in these responses.

It should also be noted that reductions in energy expenditure, activity and leptin action observed in *Myrf* ^*fl/fl*^ mice here could contribute to other aspects of their phenotype – for example, their learning and memory deficits [[8], [9], [10]]. However, since learning produces activity-dependent oligodendrogenesis in specific brain areas such as the subcortical white matter [8,9]; interruption of this local effect, rather than systemic effects originating in the ME, is perhaps more likely to underlie learning and memory dysfunctions

Our work offers new insights into how the ME couples energy sensing to adaptive responses essential for normal fuel homeostasis. We show that unlike what occurs in other brain regions [12], rapid turnover of myelinating OLs is a feature of the healthy adult ME, and that this process is required for proper sensing of fuel-related signals such as leptin. What could be the function of this energetically demanding process? Such high turnover exists in epithelial barriers where external insults continuously compromise cellular integrity [37]. Likewise, the myelinating OL population of the ME sits at the ME-ARC interface and is exposed to gluco- and lipo-toxic components of the blood that might rapidly affect the composition and structure of local myelin, perhaps forming a trigger for OL apoptosis and turnover. However, the fact that nutrient availability regulates ME myelin plasticity suggests this might form a mechanism through which the hypothalamus adapts to changes in energy levels. Therefore, future studies could determine whether continuous turnover of myelinating OLs protects the mediobasal hypothalamus from metabolic or inflammatory insults associated with obesity, type 2 diabetes, and related disorders.

## Author contributions

Conceptualization: SB, SK, RH, TS, WDR, CB. Methodology: SB, SK, RH, CB. Investigation: SB, RH, CB. Analysis: SB, CB. Visualization: SB., RH, CB. Funding acquisition: SB, WDR, CB. Writing – original draft: SB, CB. Writing – review & editing: SB, CB.

## Data Availability

Data will be made available on request.
