# Supplementary material for: Median eminence myelin continuously turns over in adult mice
Source: Mol Metab. 2023 Feb 4;69:101690. doi: 10.1016/j.molmet.2023.101690 (PMC9950957; doi:10.1016/j.molmet.2023.101690)
Supplement: Multimedia component 2 [file mmc2.docx]

**SUPPLEMENTARY FIGURES**


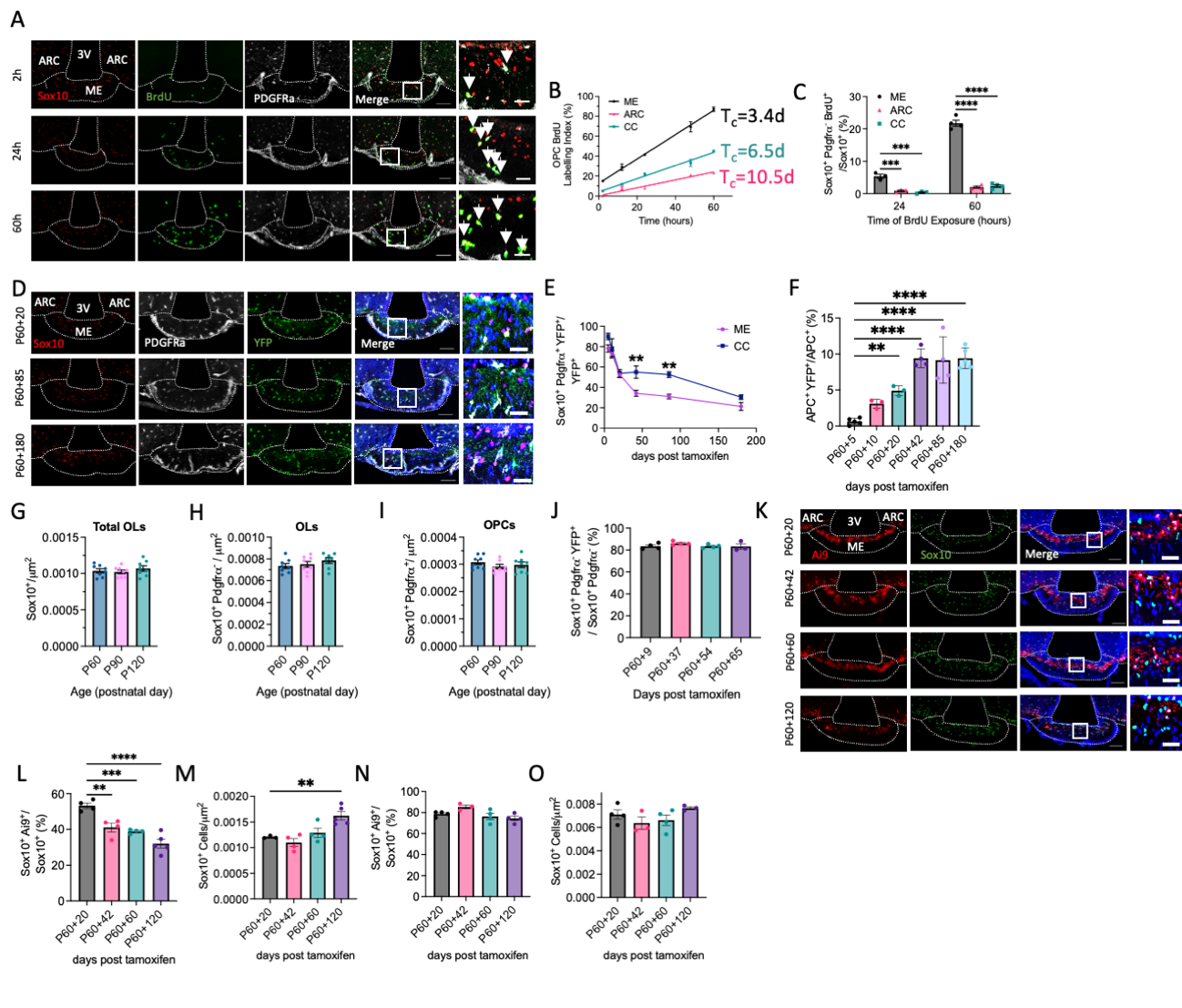


**Supplementary Figure 1. Rapid generation and turnover of oligodendrocytes in the healthy adult median eminence**

(**A**) Representative images of OL markers and BrdU in the ME of C57BL/6J mice treated with BrdU. (**B**) The proportion of proliferating OPCs over time was plotted for each region and the resulting graph used to calculate T_C_. (**C**) Quantification of OL lineage cells expressing BrdU but lacking OPC marker expression in the ME, ARC and CC of BrdU-treated C57BL/6J mice. (**D**) Representative images of the ME of *Pdgfra-Cre/ER^T2^;R26R-YFP* mice immunolabelled for YFP and OL markers and (**E**) the associated quantification of OPC marker expression in YFP+ cells over time. (**F**) Quantification of the proportion of adult born OLs generated from OPCs after P60 in the CC of *Pdgfra-Cre/ER^T2^;R26R-YFP* mice. Quantification of (**G**) OL lineage cells, (**H**) OLs and (**I**) OPCs in the ME of C57BL/6J mice at P60, P90 and P120. (**J**) Quantification of YFP-labelled OLs in the CC of *Plp-Cre/ER^T2^;R26R-eG*FP –mice following tamoxifen administration at P60. (**K**) Representative images of Ai9 expression in OLs in the ME of *Opalin-CreER^T2^;Ai9* mice following tamoxifen administration and associated quantifications (**L**,**M**). Quantification of (**N**) Ai9 reporter expression in OLs and (**O**) OLs in the CC of *Opalin-CreER^T2^;Ai9* mice. For all images, overview scale bars = 100 μm, inset scale bars = 20 μm. Data analysed by one- or two-way ANOVA with post-hoc analysis by Dunnett’s or Sidak’s multiple comparisons test, **p,0.01, ***p<0.001, ****p<0.0001, n=3-8/group.


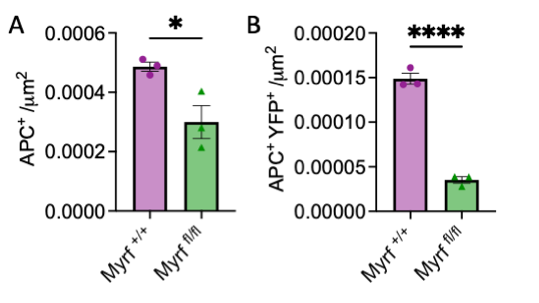


**Supplementary Figure 2. Adult-onset deletion of myelin regulatory factor blunts oligodendrogenesis in the adult median eminence**

Quantification of the density of (**A**) mature oligodendrocytes expressing APC and (**B**) adult-born oligodendrocytes in the median eminence of myelin regulatory factor knockout (*Myrf ^fl/fl^*) mice and wild-type controls (*Myrf ^+/+^)* three weeks after tamoxifen administration at P60. Analysed by student’s t-test, *p<0.05, ****p<0.0001, n=3/group.


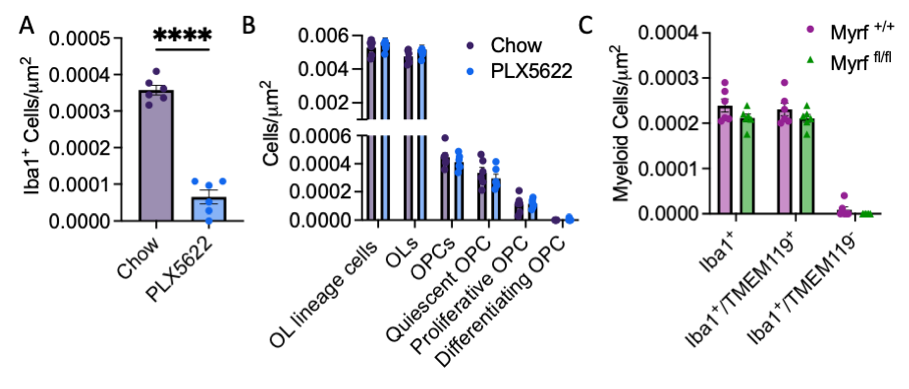


**Supplementary Figure 3. PLX5622 treatment or genetic deletion of myelin regulatory factor does not affect oligodendrocyte lineage cells in the corpus callosum**

Quantification of the density of **(A)** Iba1+ microglia and (**B**) oligodendrocyte lineage cell populations in the corpus callosum of control and PLX5622-treated C57BL/6J mice. OL subtypes were distinguished as follows; OL lineage cells = Sox10^+^, OLs = Sox10^+^/PDGFRα^-^, OPCs = Sox10^+^/PDGFRα^+^, Quiescent OPC = Sox10^+^/PDGFRα^+^/BrdU^-^, Proliferative OPC = Sox10^+^/PDGFRα^+^/BrdU^+^, Differentiating OPC = Sox10^+^/PDGFRα^-^/BrdU^+^. (**C**) Quantification of myeloid cells in the corpus callosum of myelin regulatory factor (*Myrf*) knockout mice (*Myrf ^fl/fl^*) and littermate controls (*Myrf ^+/+^*) after tamoxifen administration at P60, n=6/group.


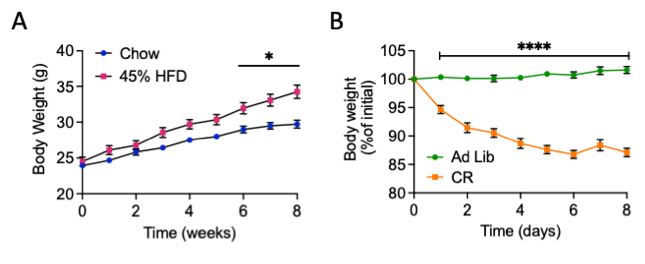


**Supplementary Figure 4. Effects of nutritional interventions on body weight**

Body weight of C57BL/6J mice fed a (**A**) control or 45% high fat diet (HFD) for 8 weeks or (**B**) chow diet ad libitum (AL) or 70% calorie restricted (CR) for 7 days. Data analysed by repeated measures two-way ANOVA, *p<0.05, ****p<0.0001, n=8/group.


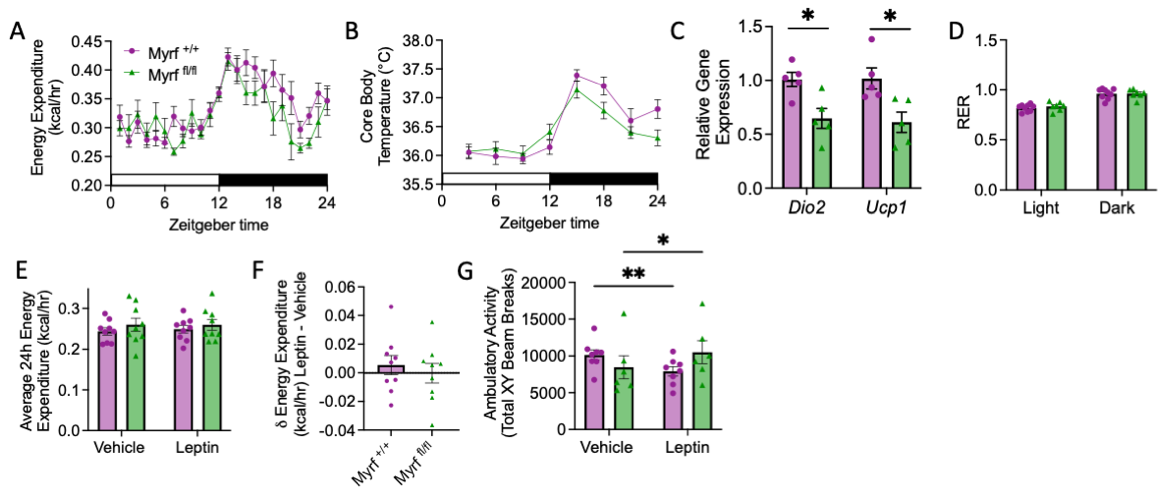


**Supplementary Figure 5. Adult born oligodendrocytes are required for the regulation of energy balance and hypothalamic leptin sensitivity**

24-hour energy expenditure (**A**) and core body temperature (**B**) of myelin regulatory factor (*Myrf*) knockout mice (*Myrf ^fl/fl^*) and littermate controls (*Myrf ^+/+^*). (**C**) Relative expression of thermogenic genes in the brown adipose tissue of *Myrf ^fl/fl^* and *Myrf ^+/+^* mice. (**D**) Light- and dark-phase respiratory exchange ratio (RER) of *ad libitum* fed *Myrf ^fl/fl^* and *Myrf ^+/+^* mice. Energy expenditure (**E-F**) and ambulatory activity (**G**) of *Myrf ^fl/fl^* and *Myrf ^+/+^* mice during a fast with vehicle or leptin administration (100 ng/hr). Data analysed by student’s t-test or two-way ANOVA with Sidak’s multiple comparisons test, *p<0.05, **p<0.01, n=6-10/group.

**Supplementary Video 1:** **Engulfment of myelin by median eminence microglia.** Video demonstrating MBP immunolabelling (green) inside Iba1^+^ microglia (red) in the median eminence of healthy, chow-fed animals.
